# Body Weight Gain Is Associated with the Disease Stage in Advanced Amyotrophic Lateral Sclerosis with Invasive Ventilation

**DOI:** 10.3390/metabo12020191

**Published:** 2022-02-19

**Authors:** Yuki Nakayama, Toshio Shimizu, Chiharu Matsuda, Michiko Haraguchi, Kentaro Hayashi, Kota Bokuda, Masahiro Nagao, Akihiro Kawata, Kazushi Takahashi

**Affiliations:** 1 Unit for Intractable Disease Nursing Care, Tokyo Metropolitan Institute of Medical Science, Tokyo 156-8506, Japan; nakayama-yk@igakuken.or.jp (Y.N.); matsuda-cr@igakuken.or.jp (C.M.); haraguchi-mc@igakuken.or.jp (M.H.); 2 Department of Neurology, Tokyo Metropolitan Neurological Hospital, Tokyo 183-0042, Japan; kentarou_hayashi@tmhp.jp (K.H.); kouta_bokuda@tmhp.jp (K.B.); masahiro_nagao@tmhp.jp (M.N.); akihiro_kawata@tmhp.jp (A.K.); kazushi_takahashi@tmhp.jp (K.T.)

**Keywords:** amyotrophic lateral sclerosis, body weight, hypometabolism, hypoalbuminemia, invasive ventilation, locked-in state

## Abstract

We investigated the incidence of weight gain and its related factors in patients with amyotrophic lateral sclerosis (ALS) who underwent tracheostomy and invasive ventilation (TIV). Seventy-eight patients with ALS and TIV were enrolled and followed up prospectively. We clarified the clinical profiles of patients with increased weight following TIV and examined chronological variations in their body mass index (BMI), energy intake, and serum albumin levels. Post follow-up, we determined their disease stage according to their communication impairment (stage I to V) and investigated factors associated with BMI increase following TIV. Patients with a post-TIV BMI increase ≥1.86 kg/m^2^ demonstrated a higher incidence of ophthalmoplegia (76.2%), total quadriplegia (61.9%), severe communication impairment (stage V; 33.3%), and hypoalbuminemia than those with a BMI increase <1.86 kg/m^2^. Patients with stage V communication impairment exhibited a larger and faster BMI decrease before TIV (mean −4.2 kg/m^2^ and −2.5 kg/m^2^/year, respectively); a larger BMI increase (mean +4.6 kg/m^2^) following TIV, despite lower energy intake; and lower albumin levels post follow-up than those with lower-stage communication impairment. Multilevel linear regression analysis demonstrated an independent association between communication impairment stages (stage V) and a post-TIV BMI increase (*p* = 0.030). Weight gain and hypoalbuminemia during TIV in patients with ALS were associated with the disease stage and may be attributable to the neurodegenerative processes that are peculiar to ALS.

## 1. Introduction

Amyotrophic lateral sclerosis (ALS) exhibits a deteriorating disease course resulting in respiratory muscle paralysis, eventually leading to death or the utilization of mechanical ventilation for survival. In Japan, the median ventilation-free survival time of patients with ALS is 4 years [1]. However, non-invasive and invasive ventilation can extend the survival time; a recent national surveillance report in Japan demonstrated that tracheostomy and invasive ventilation (TIV) prolonged the survival time by more than 10 years in patients with ALS [2].

Researchers have identified several prognostic factors for survival in patients with ALS, upon determining death or tracheostomy as the endpoints. These factors include the age at onset; body region affected at onset; diagnostic delay from onset; progression rate of the revised ALS functional rating scale (ALSFRS-R) score from onset; and respiratory, bulbar, nutritional, and psychological factors [3]. Regarding nutritional aspects, low body mass index (BMI) at diagnosis [4,5], rapid decline rate of body weight [6], and hypermetabolism [7,8,9] in the early stages predicted short survival time or an early need for tracheostomy. Recent evidence has highlighted the involvement of the hypothalamus in lesions responsible for weight loss or hypermetabolism [10].

Contrary to early-stage prognostic factors, there are limited studies on the predictive factors for functional prognosis in patients with advanced ALS undergoing TIV. The rapid progression rate from onset to TIV initiation predicts the development of severe communication impairments, including a totally locked-in state [11]. Rapid weight loss until tracheostomy predicts a functional decline during the advanced stages with long-term TIV use [12]. However, in clinical settings, we often encounter patients with ALS who show notable weight gain during TIV use. Assuming the weight loss in the early stages is the outcome of non-motor system neurodegeneration, including in the hypothalamus, weight gain or other nutritional problems in the advanced stage with TIV might also result from advanced non-motor neurodegeneration in ALS. Our hypothesis is that weight gain in the advanced stage is associated with disease progression.

This study aimed to investigate body weight variation during long-term TIV use, particularly focusing on the relationship between weight gain and hypoalbuminemia and the disease stage in patients with advanced ALS. Herein, we report that rapidly deteriorating patients with ALS and weight loss before TIV displayed significant weight gain and malnutrition during TIV use, and we discuss its pathomechanism from the perspective of hypothalamic involvement in ALS.

## 2. Results

Table 1 outlines the clinical characteristics of the enrolled 78 patients with ALS. In the total cohort of patients, there was a significant decrease in BMI at the time of TIV initiation compared with that at diagnosis and a significant increase at the end of follow-up compared with that at TIV initiation. The average increase in BMI following TIV was 1.86 kg/m^2^; thus, we first classified the patients into two groups: those with a post-TIV BMI increase <1.86 kg/m^2^ and with an increase ≥1.86 kg/m^2^. A comparison of both groups indicated a similar BMI at diagnosis. However, in the group with post-TIV BMI increase ≥1.86 kg/m^2^, the BMI at TIV use was lower, and the BMI difference from diagnosis to TIV use tended to be larger than in the group with an increase <1.86 kg/m^2^. The annual increase rate of BMI (∆BMI; see the Methods) after TIV use to the end of follow-up was also larger in the group with a post-TIV increase ≥1.86 kg/m^2^. Energy intake at TIV use and the end of follow-up was similar between both groups. However, the serum albumin levels were lower in the group with a post-TIV increase ≥1.86 kg/m^2^. The incidence of ophthalmoplegia and communication impairment was higher in the group with a post-TIV BMI increase ≥1.86 kg/m^2^ (Table 1).

Table 2 summarizes the incidence of non-motor manifestations following TIV use. The group with a post-TIV BMI increase ≥1.86 kg/m^2^ displayed a higher incidence of dysuria compared with the group with an increase <1.86 kg/m^2^, thereby necessitating a urinary catheter insertion.

**Figure 1:**
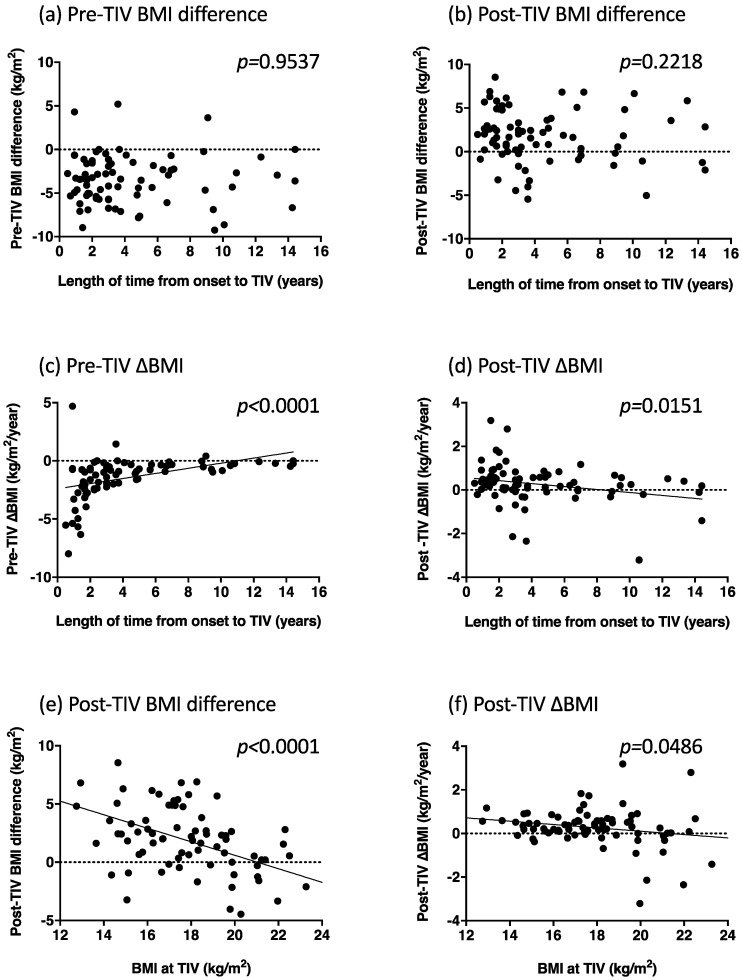
Correlations between the length of time from the onset to tracheostomy and invasive ventilation (TIV), and pre-TIV body mass index (BMI) difference (**a**), post-TIV BMI difference (**b**), pre-TIV ∆BMI (**c**), and post-TIV ∆BMI (**d**). Correlations between the BMI values at TIV use and post-TIV BMI difference (**e**) and post-TIV ∆BMI (**f**).

Pre- and post-TIV BMI differences were not correlated with the length of time from diagnosis to TIV use (*p* = 0.9537 and *p* = 0.2218, respectively; Figure 1a,b). However, pre- and post-TIV ∆BMI were correlated with the length of time from the diagnosis to TIV use (*p* < 0.0001 and *p* = 0.0151, respectively; Figure 1c,d): The shorter the time until TIV use, the greater rate of decrease and increase in pre- and post-TIV BMI, respectively. Post-TIV BMI difference and post-TIV ∆BMI were negatively correlated with BMI at TIV use (*p* < 0.0001 and *p* = 0.0486, respectively; Figure 1e,f): The lower the BMI at TIV use, the greater the post-TIV BMI increase and the rate of BMI increase. We did not observe significant correlations between the duration of TIV and BMI at the end of follow-up (*p* = 0.2182), post-TIV BMI difference (*p* = 0.3325), or post-TIV ∆BMI (*p* = 0.6664).

**Figure 2:**
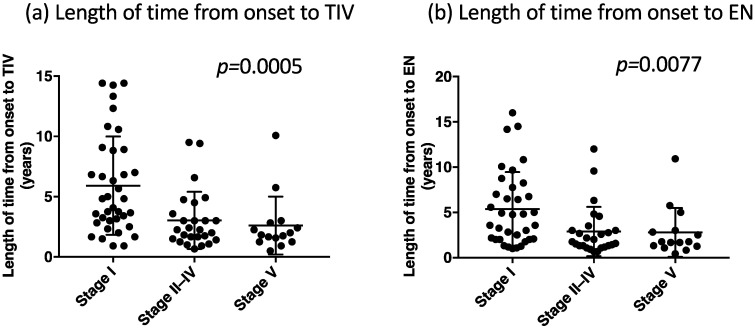
The length of time from the onset to tracheostomy and invasive ventilation (TIV) in each group classified by the communication stage (**a**) and the length of time from the onset to enteral nutrition (EN) in each group of the communication stage (**b**). *p*-Values were obtained from the one-way analysis of variance (ANOVA) between the three groups.

**Figure 3:**
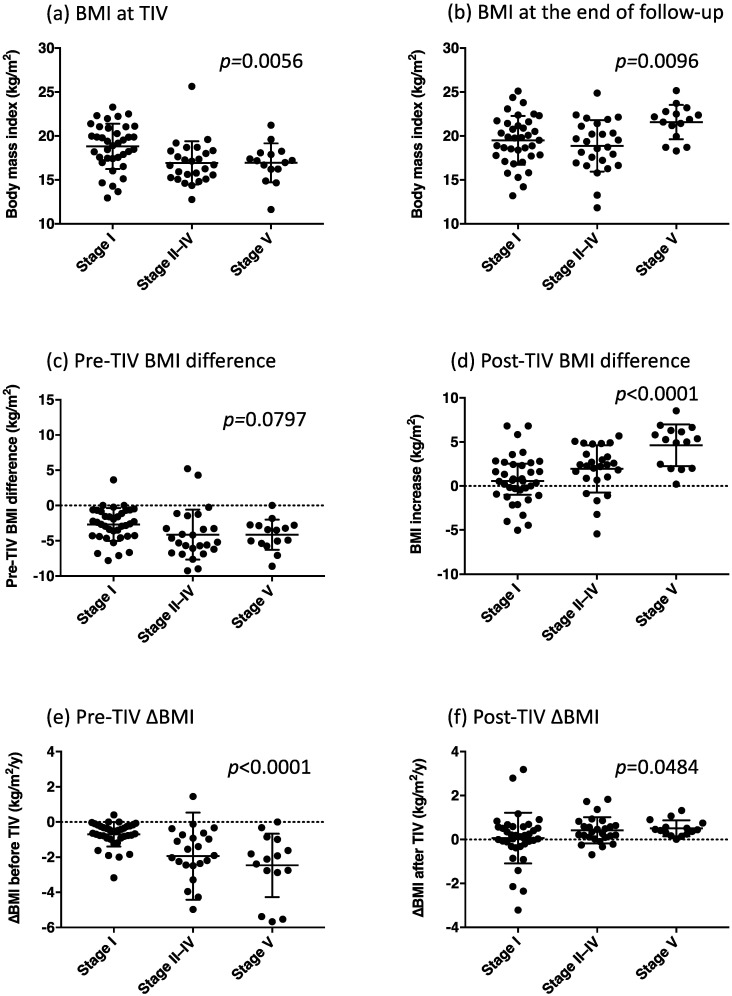
BMI parameters in each group classified by the communication stage, namely the body mass index (BMI) at tracheostomy and invasive ventilation (TIV) (**a**), BMI at the end of the follow-up (**b**), pre-TIV BMI difference (**c**), post-TIV BMI difference (**d**), pre-TIV ∆BMI (**e**), and post-TIV ∆BMI (**f**). *p*-Values were obtained from the one-way analysis of variance (ANOVA) between the three groups.

**Figure 4:**
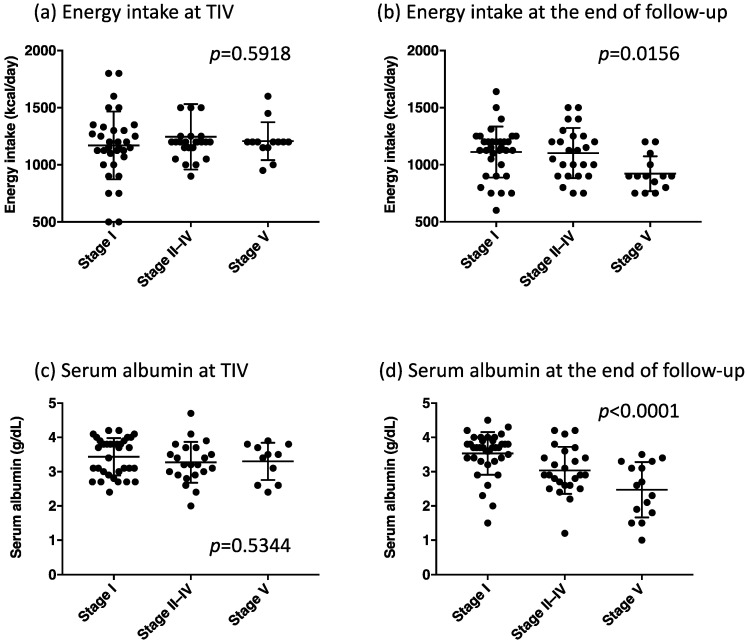
Daily energy intake and serum albumin levels in each group classified by the communication stage, namely energy intake at tracheostomy and invasive ventilation (TIV) (**a**), energy intake at the end of the follow-up (**b**), serum albumin at TIV (**c**), and serum albumin at the end of the follow-up (**d**). *p*-Values were obtained from the one-way analysis of variance (ANOVA) between the three groups.

Figure 2, Figure 3 and Figure 4 depict the clinical parameters for each communication stage at the end of follow-up (stages I, II–IV, and V). Patients who developed stage V communication impairment demonstrated the shortest length of time from the onset to TIV use and from the onset to the initiation of enteral nutrition (Figure 2a,b). Moreover, they exhibited the lowest BMI at TIV use (Figure 3a), the largest BMI at the end of follow-up (Figure 3b), a tendency of substantial BMI decrease before TIV use (Figure 3c), the largest BMI increase after TIV use (Figure 3d), the highest rate of BMI decrease before TIV (Figure 3e), and a tendency of high rate of BMI increase after TIV use (Figure 3f).

The multilevel linear regression analysis suggested that the BMI at TIV use and stage V communication impairment exerted independent significant effects on the BMI variation (coefficient 0.584, 95% CI 0.320–0.848, *p* < 0.0001, d = 0.403; Table 3). The coefficient results indicated that low BMI at TIV use and advanced communication stage were associated with increased BMI after TIV use. In particular, stage V communication impairment exerted a different effect on BMI increase compared to the other stages (coefficient 1.469, 95% CI 0.142–2.789, *p* = 0.030, d = 1.014; Table 3), indicating a significant association between BMI increase and disease progression following TIV.

Energy intake at TIV use did not differ between the stages (Figure 4a); however, patients who reached stage V had the lowest energy intake at the end of follow-up (Figure 4b). Serum albumin levels at TIV use did not differ between the stages (Figure 4c); nonetheless, patients with stage V communication impairment displayed the lowest serum albumin levels at the end of follow-up (Figure 4d). At the end of follow-up, there were no significant correlations between serum albumin levels and BMI (*p* = 0.1243), and between albumin levels and energy intake (*p* = 0.0902).

## 3. Discussion

This study investigated body weight variation during long-term TIV use, and its relationship with disease stage (communication impairment). Our findings revealed that body weight in patients with ALS increased during long-term TIV use. We also observed that weight gain following TIV was associated with an initial weight loss before TIV and the disease stage (communication impairment) during TIV use. Patients who developed the most severe communication impairment (totally locked-in state) during TIV displayed a rapid progressive weight decline until TIV, the lowest BMI at TIV use, and the largest weight gain at the advanced stage of TIV use. Notably, these patients had low energy intake and serum albumin, despite a large weight gain.

Since the skeletal muscles in patients with ALS during a long-term TIV are considered to be almost completely destroyed, the main component of the weight gain might be fat. Previous studies of energy metabolism in ALS patients reported markedly low energy expenditure in those using TIV (700 to 1000 kcal/day), indicating “hypometabolism” [13,14], which might lead to fat accumulation and weight gain. In this study, patients with the most advanced stage of communication impairment (stage V) received the lowest energy intake. However, they showed the largest increase in weight, suggesting that weight gain is pathognomonic to ALS and is related to central metabolic dysregulation.

Weight loss in the early stage of ALS is an independent prognostic factor for short survival time [4,5], and hypermetabolism specific to ALS has been suggested as weight loss pathophysiology. Metabolic analyses using indirect calorimetry [7,8,9] and the doubly labeled water method [15,16] have revealed hypermetabolism in approximately 40%–50% of patients with ALS. Furthermore, the involvement of the hypothalamus has been debated as an etiological lesion for weight loss through radiological [17,18], biochemical [19], pharmacokinetical [20,21], and neuropathological investigations [17,19,22]. The presence of TDP-43 protein inclusions in the hypothalamus suggests that ALS is a multisystem neurodegenerative disorder involving the central autonomic or metabolic nervous system [19,22,23,24].

The effects of degenerative lesions in the hypothalamus may emerge as metabolic or autonomic symptoms in the advanced stages of TIV. We observed a significant association between weight gain and dysuria in this study, suggesting a dysfunction of the central autonomic system. A previous study using indirect calorimetry in ALS reported that patients with a totally locked-in state (stage V) demonstrated a complete loss of reactive energy consumption following enteral feeding [14], suggesting a malfunction of the central metabolic regulation system. In agreement with these findings, weight gain in advanced stages can be the outcome of ALS-related central metabolic or autonomic dysregulation. We hypothesize that the central metabolic dysregulation in ALS induces “hypermetabolism” with weight loss in the early stage and “hypometabolism” with weight gain in the advanced stage of patients using TIV. Hypometabolism may lead to previously reported manifestations in advanced ALS, such as visceral fat accumulation [25], macroglossia [26], hyperosmolar hyperglycemic state [27], hypertensive attack [28], and hypothermia [29].

Hypoalbuminemia in patients with stage V disease may be attributed to multifactorial causes, such as low energy intake, long-term immobilization, and a fatty liver. However, patients with stage V ALS displayed an increased weight compared to those in the earlier stages. Furthermore, liver function test results are usually normal in patients with ALS (data not shown), with similar immobilization states between patients with stage V and those with other stages. There were no correlations between serum albumin levels and BMI or energy intake at the end of follow-up. Hence, hypoalbuminemia was possibly associated with centrally derived hypometabolism. Patients with advanced stage ALS using long-term TIV displayed an almost complete loss of skeletal muscles. Protein metabolism might also be disrupted at this stage. Furthermore, metabolic dysregulation in ALS includes a fuel shift from glucose to lipid utilization [8,30], suggesting lipids are the primary energy source in patients with advanced-stage ALS with hypoalbuminemia.

The first limitation of this study was the small sample size. Generally, the number of patients with ALS who desire to use TIV is limited. In Japan, approximately 20% of patients with ALS use TIV [2]. The 78 patients enrolled in our study might correspond to 400 patients as a background population of ALS patients; therefore, the sample size appears sufficient for studying patients with advanced ALS using TIV. The second limitation was the lack of data on total or resting energy expenditure and the volume of fat and fat-free mass, which warranted the evaluation of fat accumulation and its relationship to BMI. The third limitation was the lack of data on metabolites related to glucose and lipid metabolism. These data would contribute to the elucidation of the fuel shift in advanced ALS. Fourthly, we should explore autonomic nervous function during TIV use to elucidate the mechanism of hypometabolism.

**Figure 5:**
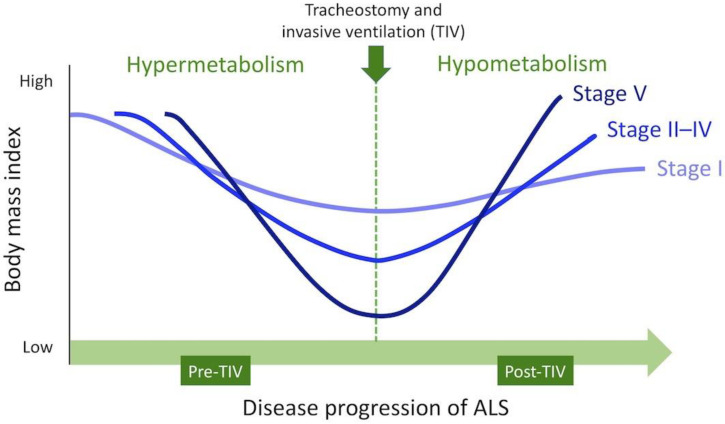
Hypothetical schema of weight variations in patients with amyotrophic lateral sclerosis. “Stage” indicates the stage of communication impairment assessed at the end of follow-up.

Figure 5 shows the hypothetical schema of weight variations of patients with ALS, classified according to the stage of communication impairment. Weight variations through clinical courses of ALS might be determined by the pathophysiology of ALS and disease progression in individual patients. Weight gain under TIV induces fat accumulation and should not be interpreted as disease stability. Clinicians usually reduce energy intake in response to progressive weight gain, resulting in malnutrition and susceptibility to infections. The reduction of energy intake to <700 kcal/day also creates an ethical issue. Appropriate nutritional therapy must be established in the future for patients with advanced ALS using TIV.

## 4. Subjects and Methods

### 4.1. Patients

**Figure 6:**
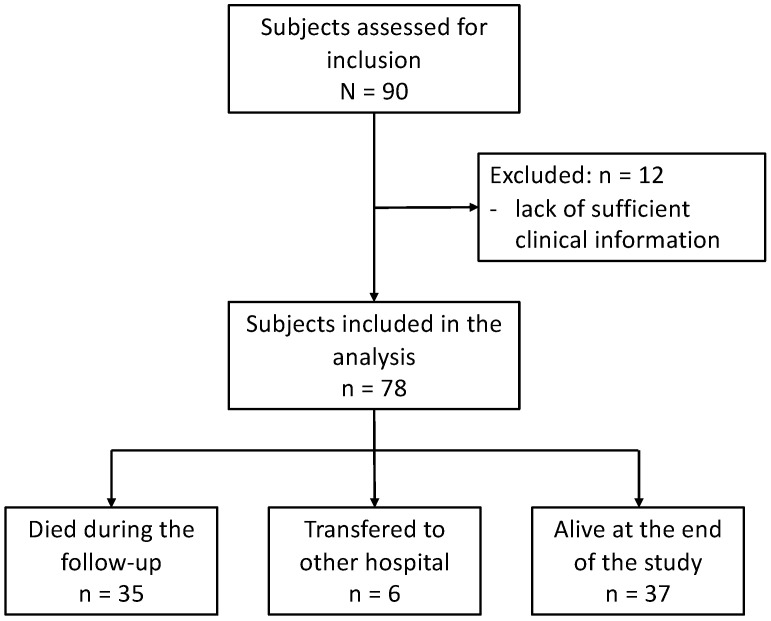
Flowchart of patient enrollment and study sample.

A total of 90 patients with sporadic or familial ALS or progressive muscular atrophy (PMA) were enrolled in the single-center, prospective observational study. The diagnosis of ALS was based on the revised El Escorial criteria, and patients with “clinically definite”, “clinically probable”, “clinically probable laboratory supported”, or “clinically possible” ALS were included [31]. We also included patients with PMA who showed no upper motor neuron signs, as PMA is considered to be equivalent to ALS [32,33]. All patients underwent TIV and were regularly followed up via home visits or regular admission to the Tokyo Metropolitan Neurological Hospital, Tokyo, Japan, between 2005 and 2017. We included patients who had already been using TIV before 2005 and those who began using TIV after 2005. Of the 90 patients, we excluded 12 patients during the follow-up due to incomplete assessment and lack of sufficient clinical information, especially body weight value. We eventually enrolled 78 patients (Figure 6); 4 patients had a family history of ALS, whereas 3 patients had superoxide dismutase 1 gene variants. We evaluated ALS-related genes only when the examination was possible, owing to clinical implications and consent provision by the patients or their families. All patients manifested a persistent and progressive disease course both before and after TIV use, besides displaying severe generalized muscle atrophy of the extremities and tongue. No other diseases affecting the nervous system were identified during follow-up. 35 patients died during follow-up, and 6 patients were transferred to other hospitals (Figure 6). The follow-up lasted until December 2019, and 37 patients were alive at the end of the study.

### 4.2. Assessment

We designed the study to answer the following three questions: (1) do patients with ALS show significant weight gain during long-term TIV use; (2) are there differences in clinical findings, including motor and non-motor deficits, between patients with larger and smaller weight gain; (3) what are the correlative factors of weight gain during TIV use, particularly when focusing on weight changes, communication ability, and serum albumin levels before and after TIV use?

Neurologists and nurses evaluated the neurological status, functional motor deficits, nutritional state, and communication ability (communication impairment stage) of patients with ALS during home visits or regular admission to our hospital. Clinical data before 2005 were retrospectively collected from the medical database for patients who had been using TIV before the study. None of the 78 patients exhibited obvious dementia during routine neurological examinations throughout follow-up.

For each patient, we evaluated the following factors: sex, the age at onset, the body region affected at onset, height (m), length of time (years) from the onset to initiation of TIV, and length of time (years) from TIV to the end of follow-up or death. To assess nutritional status, we evaluated the body weight (kg) at the time of diagnosis, at TIV initiation, and at the end of follow-up (final evaluation). Furthermore, we calculated the annual rate of BMI variation (∆BMI, kg/m^2^/year) from diagnosis to TIV use, and from TIV use to the final evaluation time or death [6,12]. The definitions of each BMI parameter are as follows:

- Pre-TIV BMI difference (kg/m^2^) = BMI at TIV use − BMI at diagnosis;
- Pre-TIV ∆BMI (kg/m^2^/year) = (BMI at TIV use − BMI at diagnosis)/time interval (years);
- Post-TIV BMI difference (kg/m^2^) = (BMI at final evaluation − BMI at TIV use);
- Post-TIV ∆BMI (kg/m^2^/year) = (BMI at final evaluation − BMI at TIV use)/time interval (years).

We also assessed the length of time from the onset to the initiation of enteral nutrition via nasogastric or gastrostomy tube, and the total duration of enteral nutrition until final evaluation time or death. Energy intake (kcal/day) via the feeding tube and serum albumin levels (g/dL) were also evaluated at TIV initiation and at the final evaluation time.

We determined communication ability to assess disease progression following the initiation of TIV [11,34]. The communication stage was defined using an augmentative and alternative communication device and was classified into five stages as follows: Stage I, communicated using sentences; stage II, one-word answers only; stage III, non-verbal yes/no responses; stage IV, occasional non-verbal yes/no response; and stage V, unable to communicate by any means. This staging method can be easily applied to patients with TIV and severely deteriorating motor functions that cannot be entirely assessed using conventional grading scales, such as the revised ALS functional rating scale (ALSFRS-R) [35]. Stage V corresponds to a totally locked-in state, characterized by the loss of voluntary movements, including ocular movements [11,36]. The communication stage was prospectively and regularly assessed at least every 3 months following TIV initiation. In addition, we compared the nutritional parameters, including BMI, ∆BMI, and serum albumin level, between the three subgroups classified by the communication stage at the final evaluation time (stage I, II–IV, and V).

Furthermore, we evaluated other motor disabilities, namely obvious oculomotor limitations and total quadriplegia. Obvious oculomotor limitations (ophthalmoplegia) were defined as persistent abnormal ocular movements during a bedside examination by at least two neurologists [11]. The evaluation of ocular movements included examinations of slow pursuit movement and ocular saccade speed with the vertical and horizontal gaze. Vertical ophthalmoplegia and slow saccade (slow eye movement) were the most common abnormalities [37,38]. Total quadriplegia indicated a failure to demonstrate visible muscle movements of the limbs. We defined the development period of the manifestations mentioned above during the follow-up for each patient [12].

We investigated the incidence of non-motor manifestations during follow-up, namely macroglossia, unstable blood pressure, hypothermia, dysuria (urinary catheter placement), and hyperglycemic state [27]. Macroglossia in patients with advanced ALS has been previously described [24,39]. We defined it as a condition characterized by the enlargement of the tongue and its constant protrusion over the dentition. Moreover, the patient could not volitionally retract the tongue into the oral cavity. Unstable blood pressure was defined as paroxysmal hypertension with a systolic blood pressure >180 mmHg, often accompanied by tachycardia (>100 bpm), which might result from central sympathetic hyperactivity [26]. Nocturnal hypotension occasionally followed daytime hypertension, whereas hypertensive attacks usually lasted for days or weeks and often recurred [26]. Hypothermia was defined as a constant body temperature <36 °C [27]. Dysuria indicated voiding difficulties that necessitated the insertion of a urinary catheter. Its causes (pelvic floor muscle weakness or neurogenic bladder) were not completely identified; however, we virtually accepted any reason for the performance of urinary catheterization in individual patients [27]. A hyperglycemic state was defined as an episode of paroxysmal hyperglycemia with a blood glucose level >400 mg/dL [25].

### 4.3. Statistical Analysis

We performed Welch’s t-test or chi-square test for data comparison between the two groups (patients with BMI increase ≥1.86 kg/m^2^ and those with BMI increase <1.86 kg/m^2^ following TIV; Table 1 and Table 2) and Pearson’s correlation coefficient for correlation analysis (Figure 1). We conducted a paired t-test for paired data (BMI variations from the time of diagnosis to TIV use and from the time of TIV use to the end of follow-up; Table 1). Comparisons between the three groups of communication stages were performed using the one-way analysis of variance (ANOVA; Figure 2, Figure 3 and Figure 4). To clarify factors related to BMI increase following TIV, we assessed the post-TIV BMI difference by multilevel linear regression analysis, including the BMI as random effects, and the time of assessment as fixed effects (Table 3). We adopted the panel data format in which the same person could appear three to six times (data from 6 years after TIV initiation) to utilize information on cessation cases during follow-up. We calculated the within-subject effect size (estimated regression coefficient divided by estimated level 1 standard deviation) for the BMI value. The effect size was considered low if d-values varied by 0.20, medium at 0.50, and large if greater than 0.80. The model included sex, age at onset, the length of time from onset to TIV use, BMI at TIV use, and communication stage at the end of follow-up as independent variables.

Statistical analysis was two-sided, and the statistical significance was set at *p* < 0.05. The analyses were performed using JMP^®^ for Macintosh version 13.0.0 (SAS Institute, Cary, NC, USA), and the multilevel linear regression analysis was conducted using STATA^®^ version 16.1 (StataCorp, Texas, TX, USA).

## Figures and Tables

**Table 1:** Clinical characteristics of all patients.

	All	Groups by BMI Increase Following TIV		*p*-Value
		BMI Increase <1.86	BMI Increase ≥1.86	
Number of patients (%)	78	37 (47.4%)	41 (52.6%)	
Sex (male) (n, %)	48 (61.5%)	20 (56.8%)	27 (65.9%)	0.4095
Age at onset (year)	56.5 (11.3)	55.2 (11.8)	57.6 (11.0)	0.3572
Onset region (bulbar) (n, %)	18 (23.1%)	7 (18.9%)	11 (26.8%)	0.4058
Number of deaths (%)	35 (44.9%)	12 (32.4%)	23 (56.1%)	0.0969
Time from onset to TIV use (years)	4.3 (3.6)	4.9 (3.6)	3.8 (3.5)	0.1967
Time from TIV use to the end of follow-up (years)	7.9 (6.2)	7.4 (7.1)	8.3 (5.2)	0.5123
Time from onset to enteral nutrition (year)	4.0 (3.6)	4.7 (3.8)	3.4 (3.3)	0.1067
Duration of enteral nutrition at the end of follow-up (year)	8.2 (5.9)	7.7 (6.6)	8.7 (5.4)	0.4373
BMI at diagnosis (kg/m^2^)	21.3 (2.5)	21.7 (2.5)	20.9 (2.5)	0.1440
BMI at TIV use (kg/m^2^)	17.8 (2.6) **	18.9 (2.7) **	16.9 (2.1) **	0.0007
BMI at the end of follow-up (kg/m^2^)	19.7 (2.8) **	18.2 (2.8) *	21.0 (2.2) **	<0.0001
BMI difference from diagnosis to TIV use (kg/m^2^)	−3.5 (2.8)	−2.9 (3.0)	−4.0 (2.5)	0.0769
∆BMI from diagnosis to TIV use (kg/m^2^/year)	−1.4 (1.8)	−1.1 (1.8)	−1.7 (1.8)	0.1533
BMI difference from TIV use to the end of follow-up (kg/m^2^)	1.9 (3.0)	−0.6 (2.0)	4.1 (1.8)	<0.0001
∆BMI after TIV use to the end of follow-up (kg/m^2^)	0.3 (0.9)	−0.2 (1.0)	0.7 (0.5)	<0.0001
Energy intake at TIV use (kcal/day)	1203 (272)	1190 (335)	1215 (201)	0.7117
Energy intake at the end of follow-up (kcal/day)	1071 (220)	1090 (244)	1055 (198)	0.5078
Serum albumin at TIV use (g/dL)	3.4 (0.6)	3.4 (0.6)	3.3 (0.5)	0.8726
Serum albumin at the end of follow-up (g/dL)	3.2 (0.8)	3.4 (0.7)	3.0 (0.8)	0.0185
Ophthalmoplegia (n, %)	48 (38.5%)	16 (44.4%)	32 (76.2%)	0.0014
Total quadriplegia (n, %)	40 (31.2%)	15 (40.5%)	25 (61.9%)	0.0662
Disabilities at the end of follow-up (n, %)				
Communication stage I	37 (47.4%)	26 (72.2%)	11 (26.2%)	<0.0001
Communication stage II–IV	26 (33.3%)	10 (27.0%)	16 (39.0%)	
Communication stage V	15 (19.2%)	1 (2.8%)	14 (33.3%)	

Values are expressed as number (%) or mean (SD). *p*-Values were obtained by comparing the values of the groups by using a chi-square test or Welch’s t-test. BMI at TIV use was compared with the BMI at diagnosis, and that at the end of follow-up was compared with the BMI at TIV use, using a paired t-test (** *p* < 0.0001 and * *p* = 0.0305). TIV: tracheostomy and invasive ventilation, BMI: body mass index, and ∆BMI: BMI decline rate per year.

**Table 2:** Frequency of non-motor manifestations.

	All (N = 78)	Groups by BMI Increase Following TIV		*p*-Value
		BMI Increase <1.86 (n = 37)	BMI Increase ≥1.86 (n = 41)	
Unstable blood pressure	20 (25.6%)	8 (22.2%)	12 (28.6%)	0.5257
Hypothermia	15 (19.2%)	4 (11.1%)	11 (26.2%)	0.0865
Dysuria (uretheral catheter insertion)	33 (42.3%)	11 (30.6%)	22 (52.4%)	0.0499
Macroglossia	16 (20.5%)	7 (19.4%)	9 (21.4%)	0.8351
Hyperglycemic state	11 (14.1%)	6 (16.7%)	5 (11.9%)	0.5418

Values are expressed as number (%). *p*-Values were obtained by comparing the values of the groups by using a chi-square test. TIV: tracheostomy and invasive ventilation, BMI: Body mass index, and ∆BMI: BMI decline rate/year.

**Table 3:** Multilevel linear regression analysis for BMI increase following TIV use.

Variables	Coefficient	95% CI	*p*-Value
Age at onset	0.002	−0.535, 0.577	0.941
Length of time from onset to TIV use	0.144	−0.184, 0.307	0.082
BMI at TIV use	0.584	0.320, 0.848	<0.0001
Stage			
Stage II	0.463	−0.368, 1.294	0.275
Stage III	0.341	−0.767, 1.449	0.547
Stage IV	0.814	−0.260, 1.888	0.138
Stage V	1.469	0.142, 2.798	0.030
Random effect			
Residual	2.099		
Cons	3.140		

BMI: body mass index, EN: enteral nutrition, TIV: tracheostomy and invasive ventilation. Patients with amyotrophic lateral sclerosis were included as random effects.

## Data Availability

Anonymized data not published within the article will be shared upon reasonable requests from a qualified investigator. The reuse of the data requires permission from the authors.
